# Assessment of all-ceramic systems' influence on dual-cure resin cement polymerization: An *in vitro* study

**DOI:** 10.6026/973206300213431

**Published:** 2025-10-31

**Authors:** Savitha P.N., Ravi Shankar Yadav, Sunayana Priyadarshini, Alok Dwivedi, Hemchand Surapaneni, Palak Joshi

**Affiliations:** 1 Department of Prosthodontics and Crown & Bridge, The Oxford Dental College, Bengaluru, Karnataka, India; 2 Department of Prosthodontics and Crown & Bridge, Dental College Azamgarh, Azamgarh, Uttar Pradesh, India; 3 Department of Prosthodontics and Crown & Bridge, Hi-tech Dental College & Hospital, Bhubaneswar, Odisha, India; 4 Faculty of Dentistry, Najran University, Najran, Kingdom of Saudi Arabia; 5 Department of Prosthodontics and Crown & Bridge, Drs Sudha & Nageswara Rao Siddhartha Institute of Dental Sciences, Chinoutpalli, Gannavaram Mandal, Andhra Pradesh, India; 6 Department of Prosthodontics and Crown & Bridge, Modern Dental College and Research Centre, Indore, Madhya Pradesh, India

**Keywords:** All-ceramic systems, dual-cure resin cement, degree of conversion, microhardness, bond strength, polymerization

## Abstract

The polymerization of dual-cure resin cements is critical for the long-term success of all-ceramic restorations is of interest. In this *in vitro* study, sixty ceramic discs were fabricated from lithium disilicate, zirconia-reinforced lithium silicate, monolithic zirconia, feldspathic porcelain and polymer-infiltrated ceramic network (n=12/group). Dual-cure resin cement was light-cured through each disc and the degree of conversion; Vickers microhardness and bond strength were measured using FTIR, microhardness testing and a universal testing machine. One-way ANOVA revealed significant differences among groups (p<0.001), with lithium disilicate showing the highest values and monolithic zirconia the lowest. Thus, we show that the type of ceramic system significantly influences resin cement polymerization, emphasizing lithium disilicate as the most favorable and monolithic zirconia as the least.

## Background:

All-ceramic restorations have been in high usage in modern-day dentistry because of their high aesthetic, biocompatibility and mechanical properties [1]. The success and the long life of such restorations are not only dependent on the ceramic material but also on the bonding procedure, the adhesive luting [2]. Luting all-ceramic restorations with dual-cure resin cement is a common practice because, unlike light-curing and self-curing, dual-cure resin cement provides sufficient polymerization even in those regions where light-curing mechanisms have limited accessibility [3]. Polymerization of resin cements is a critical measure since the material may not cure well, hence giving low mechanical properties, low bond strength, high solubility and may fail altogether [4]. Polymerization of dual-cure resin cement is affected by a number of factors, such as the light intensity, exposure period, resin cement composition and the nature and thickness of overlying restoration [5]. The most important of them is the translucency and optical properties of the ceramic system, which define how much light penetrates to the underlying resin cement [6]. New developments in dental ceramics have presented numerous all-ceramic systems, including lithium disilicate (LDS), zirconia-reinforced lithium silicate (ZLS), monolithic zirconia (MZ), feldspathic porcelain (FP) and polymer-infiltrated ceramic network (PICN) [7]. The systems have different optical and structural properties that could have different influences on the polymerization of resin cements. As an example, ceramics with high transparency, such as LDS and FP, transmit more light than more opaque ceramics, such as MZ and could lead to higher conversion degrees and better mechanical properties of the resin cement [8]. The influence of ceramic thickness and translucency on polymerization of resin cements has been examined in several studies with inconclusive findings [9, 10]. Other authors did notice important variations in the level of conversion and microhardness of resin cements when cured using various ceramic types [11], whereas others did not find any important differences [12]. In addition, the majority of investigations were conducted in a narrow scope of ceramic systems or one characteristic (*e.g.*, level of conversion) without studying several parameters simultaneously (*e.g.*, microhardness, bond strength). Although it is clinically relevant, modern all-ceramic systems and their effects on the polymerization of dual-cure resin cements are not unanimously agreed upon. The existence of this knowledge gap explains why it is important to undertake a systematic *in vitro* analysis of the effects of different ceramic systems on the properties of dual-cure resin cements of interest. Therefore, it is of interest to evaluate the effect of five various all-ceramic systems on the extent of conversion, Vickers microhardness and bond strength of dual-cure resin cement.

## Materials and Methods:

## Study design:

This *in vitro* study was designed to evaluate the effect of five different all-ceramic systems on the polymerization of dual-cure resin cement. The experimental groups were defined by the type of all-ceramic system used: lithium disilicate (LDS), zirconia-reinforced lithium silicate (ZLS), monolithic zirconia (MZ), feldspathic porcelain (FP) and polymer-infiltrated ceramic network (PICN). The assessed outcomes were degree of conversion (DC), Vickers microhardness (VMH) and bond strength (BS).

## Sample size calculation:

Based on a pilot study, an effect size of 0.8 was assumed for DC among groups. Using G*Power software (version 3.1.9.4, Heinrich-Heine- Universität Düsseldorf, Germany), with α=0.05, power (1-β)=0.80 and five groups, a total sample size of 45 was calculated (n=9 per group). To account for potential technical errors and increase reliability, the sample size was increased to 12 per group (total N=60).

## Materials:

The all-ceramic systems and dual-cure resin cement used in this study are detailed in Table 1 (see PDF). All materials were used according to the manufacturer's instructions.

## Specimen preparation:

Ceramic discs (10 mm diameter, 1.5 mm thickness) were fabricated from each all-ceramic system (n=12 per group). For LDS and ZLS, CAD/CAM blocks were sectioned using a low-speed diamond saw (Isomet, Buehler, Lake Bluff, IL, USA) and crystallized/fired as per manufacturer recommendations. MZ discs were milled from pre-sintered zirconia blanks and sintered at 1450°C for 2 hours. FP discs were fabricated by layering porcelain on a refractory die and firing according to the manufacturer's cycle. PICN discs were cut from CAD/CAM blocks and finished as per instructions. All discs were polished with 600-, 800- and 1200-grit silicon carbide paper under water cooling to achieve a standardized thickness and smooth surface. Thickness was verified with a digital caliper (Mitutoyo, Tokyo, Japan) with an accuracy of 0.01 mm.

## Resin cements polymerization:

Dual-cure resin cement (Variolink N, IvoclarVivadent, Schaan, Liechtenstein) was mixed according to the manufacturer's instructions. The cement was applied to a mylar strip and covered with another mylar strip. Each ceramic disc was placed on top and a 1 kg load was applied for 10 seconds to ensure uniform thickness (0.1 mm). The resin cement was light-cured through the ceramic disc using an LED curing unit (Bluephase G2, IvoclarVivadent) with an irradiance of 1200 mW/cm^2^ for 20 seconds on each side (total 40 seconds). The irradiance was verified with a radiometer (Bluephase Meter, IvoclarVivadent) before each curing session. After polymerization, specimens were stored in distilled water at 37°C for 24 hours before testing.

## Degree of conversion (DC) measurement:

DC was assessed using Fourier transform infrared spectroscopy (FTIR) (Spectrum Two, PerkinElmer, Waltham, MA, USA). For each specimen, the absorbance peaks of the methacrylate groups (1636 cm-^1^) and the internal standard (aromatic C-C, 1608 cm-^1^) were recorded before and after polymerization. DC was calculated using the formula:

DC (%) = [1 - (A1636/A1608)p / (A1636/A1608)u] x 100

Where A is the absorbance peak height, p is the polymerized resin and u is the unpolymerized resin. Three measurements were taken for each specimen and the mean value was recorded.

## Vickers microhardness (VMH) measurement:

VMH was measured using a microhardness tester (HMV-G21, Shimadzu, Kyoto, Japan). A 50 g load was applied for 15 seconds. Three indentations were made on the surface of each resin cement specimen and the mean value (in Vickers hardness number, VHN) was calculated.

## Bond strength (BS) measurement:

BS was evaluated using a universal testing machine (Instron 5966, Instron, Norwood, MA, USA). Resin cement was bonded to composite resin discs (10 mm diameter, 2 mm thickness) simulating tooth structure, using the same ceramic discs as intermediates. After 24 hours of storage in distilled water at 37°C, the specimens were subjected to shear bond strength testing at a crosshead speed of 1 mm/min. The maximum load at failure (in MPa) was recorded.

## Statistical analysis:

Data were analyzed using SPSS software (version 25.0, IBM, Armonk, NY, USA). Normality was assessed using the Shapiro-Wilk test and homogeneity of variances was verified with Levene's test. One-way ANOVA was used to compare DC, VMH and BS among the five groups. Post-hoc multiple comparisons were performed using Tukey's test. The significance level was set at α=0.05.

## Results:

Descriptive statistics for DC, VMH and BS for all groups are presented in Tables 1 (see PDF), 2 (see PDF) and 3 (see PDF), respectively. Significant differences were observed among the five all-ceramic systems for all evaluated parameters (p<0.001). The highest DC was observed in the LDS group (68.4±2.1%), followed by ZLS (62.7±1.8%), FP (60.3±2.0%), PICN (55.1±1.7%) and MZ (48.3±1.9%). Post-hoc analysis revealed that all groups were significantly different from each other (p<0.05), except for the comparison between ZLS and FP (p=0.082). VMH values followed a similar trend to DC. The LDS group exhibited the highest VMH (54.2±1.8 VHN), while the MZ group showed the lowest (38.7±1.5 VHN). Intermediate values were found for ZLS (50.3±1.6 VHN), FP (48.1±1.7 VHN) and PICN (43.5±1.4 VHN). All pairwise comparisons were statistically significant (p<0.05). The BS values ranged from 18.2±1.2 MPa (MZ) to 28.7±1.5 MPa (LDS). The ZLS and FP groups showed comparable BS (25.4±1.3 MPa and 24.8±1.4 MPa, respectively; p=0.152), while PICN had a significantly lower BS (21.3±1.1 MPa) than ZLS and FP but higher than MZ (p<0.05).

## Discussion:

This study evaluated the influence of five all-ceramic systems on the polymerization of dual-cure resin cement by assessing DC, VMH and BS. The results demonstrated that the type of all-ceramic system significantly affects all evaluated properties, leading to rejection of the null hypothesis. The LDS group consistently exhibited the highest DC, VMH and BS, while the MZ group showed the lowest values. These findings highlight the critical role of ceramic translucency and composition in resin cement polymerization [13]. The degree of conversion is a key indicator of resin cement polymerization, as it reflects the percentage of unreacted methacrylate groups converted to polymer [14]. In the present study, DC varied from 48.3% (MZ) to 68.4% (LDS), which is consistent with previous reports [15]. The superior DC observed with LDS can be attributed to its high translucency, which allows greater light transmission compared to more opaque ceramics like MZ [16, 17]. ZLS and FP showed intermediate DC values, with no significant difference between them. ZLS combines the aesthetics of glass-ceramics with the strength of zirconia, but its translucency is slightly lower than LDS due to the zirconia content [18]. FP, despite being a glass-ceramic, may have slightly lower light transmission than LDS due to differences in microstructure and crystalline content [19]. PICN, a hybrid material with a polymer network, demonstrated lower DC than LDS, ZLS and FP, likely due to light scattering within its polymer-ceramic structure [20]. MZ, being a polycrystalline ceramic with high opacity, exhibited the lowest DC, which aligns with the findings of previous studies [21]. Vickers microhardness is an indirect measure of the degree of polymerization and cross-linking density of resin cements [22]. The VMH values in this study followed the same trend as DC, with LDS showing the highest hardness and MZ the lowest. This correlation between DC and VMH is expected, as a higher degree of conversion typically results in a more densely cross-linked polymer network, leading to increased hardness [23]. The VMH values observed in this study (38.7-54.2 VHN) are within the range reported in the literature for dual-cure resin cements [24]. The significant differences in VMH among groups further emphasize the impact of ceramic type on resin cement polymerization.

The strength of bonds is a vital parameter in the clinical success of all-ceramic restorations, because it guarantees retention and marginal seal [25]. In this research, BS was found to be 18.2 to 28.7 MPa (MZ and LDS, respectively) with all falling above the 17-20 MPa minimal acceptable bond strength in clinical use [26]. The high bond strength as observed with LDS is owed to the high translucency, which enables sufficient light activation of the resin cement that leads to better polymerization and adhesion strength [27, 28]. The clinical implication of this research is huge. Clinicians need to know that all-ceramic system selection may affect polymerization of dual-cure resin cements, especially with opaque resin cements such as MZ. Prolongation of the curing period, high-intensity light-curing devices, or the use of self-cure activators might be required in these situations to have sufficient polymerization [29]. Also, the use of transparent ceramics such as LDS can be beneficial where maximum resin cement polymerization is vital, such as high-stress points or thick restorations. There are limitations to this study. First, it was performed *in vitro* and clinical conditions (*e.g.*, moisture, temperature changes and oral activity) might play a role. Second, there was a single assessment of a dual-cure resin cement; not all cements are the same in reaction to different ceramic systems. Third, the ceramic thickness was standardized at 1.5 mm, which is not necessarily all clinical situations. The impact of varying ceramic thicknesses, as well as the performance of various resin cements, should be studied in the future. Finally, the nature of the all-ceramic system is largely going to affect the polymerization of the dual-cure resin cement, with LDS showing the most appropriate characteristics and MZ the worst ones. These results emphasize that ceramic translucency and composition need to be taken into account when choosing resin cements and curing regimes to use with all-ceramic restorations.

## Conclusion:

This *in vitro* experiment established that the nature of the all-ceramic system has a tremendous influence on the polymerization of the dual-cure resin cement. Lithium disilicate (LDS) produced the highest conversion, Vickers microhardness and bond strength and monolithic zirconia (MZ) produced the lowest values. Zirconia-reinforced lithium silicate (ZLS), feldspathic porcelain (FP) and polymer-infiltrated ceramic network (PICN) had intermediate properties.

## References

[1] Majumder A (2019). J Indian Prosthodont Soc..

[2] Caprak YO (2019). J Prosthodont..

[3] Turp V (2018). J Esthet Restor Dent..

[4] Borges LPS (2021). J Mater Sci Mater Med..

[5] Zhang X, Wang F. (2011). Chin Med J (Engl)..

[6] Pishevar L (2019). J Contemp Dent Pract..

[7] Cho SH (2015). J Contemp Dent Pract..

[8] Kumari S (2021). J Contemp Dent Pract..

[9] Turp V (2015). J Istanb Univ Fac Dent..

[10] Lopes Cde C (2015). Braz Dent J..

[11] Kilinc E (2011). Oper Dent..

[12] Moreno MBP (2018). Braz Dent J..

[13] Hooshmand T (2009). J Prosthodont..

[14] Pick B (2010). Eur J Dent..

[15] AlShaafi MM (2014). J Adhes Dent..

[16] Öztürk E (2015). Acta Odontol Scand..

[17] Gregor L (2014). J Prosthet Dent..

[18] Turp V (2011). J Prosthodont..

[19] Bansal R (2016). J Conserv Dent..

[20] Borges GA (2008). Oper Dent..

[21] da Costa Neto D (2017). Eur J Prosthodont Restor Dent..

[22] Samimi P (2018). J Dent (Tehran)..

[23] Valentino TA (2010). Braz Dent J..

[24] Watanabe H (2015). Oper Dent..

[25] Öztürk E (2013). J Dent..

[26] Zhang L (2019). J Prosthodont..

[27] Turkoglu P, Sen D. (2019). Biomed Res Int..

[28] Ansarifard E (2021). J Indian Prosthodont Soc..

[29] Gultekin P (2015). J Istanb Univ Fac Dent..

